# Rural multidimensional poverty and livelihood mix: A micro level study in Bihar, India

**DOI:** 10.1016/j.heliyon.2025.e42772

**Published:** 2025-02-18

**Authors:** Ogbonna Amarachi Onyeyirichi, M.G. Deepika

**Affiliations:** a Amrita School for Sustainable Futures, Amrita Vishwa Vidyapeetham, Amritapuri, India; b Department of Economics, Alliance University, Bangalore, India

**Keywords:** Rural multidimensional poverty, Access to livelihood capitals, Livelihood mix, Livelihood diversification, Sustainable livelihoods, India

## Abstract

Understanding the livelihood dynamics of rural households is crucial for formulating rural, pro-poor policies. Quite a few studies in the past have explored the impact of several aspects of livelihoods on rural poverty using unidimensional or multidimensional poverty measures, which fail to capture the nuances of rural poverty sufficiently. Identifying the gap in the literature, we explore the dynamics of rural poverty in relation to access to livelihood capitals and livelihood diversification of the rural households in India. We measure the rural poverty using the Rural multidimensional poverty index (RMPI) developed by FAO and OPHI (Oxford Poverty and Human Development Initiative) in 2022, access to livelihood capitals using the Alkire-Foster method and livelihood diversification using the Simpson's Diversification Index (SDI). We examine whether livelihood diversification mediates the relationship between access to livelihood capitals and rural multidimensional poverty, as suggested by the Sustainable Livelihood Framework. Our results show that there is low livelihood diversification and lack of access to livelihood capitals in our study area. We also find that livelihood diversification does not mediate the relationship between access to livelihood capitals and rural multidimensional poverty, but access to livelihood capitals is instrumental for poverty reduction in rural communities.

## 1 Introduction

Poverty and livelihood are multifarious, location-specific issues intertwined and dire in the rural third-world context [[1], [2], [3]]. Recent studies establish that households’ livelihoods determine their poverty status [4,5], especially in the rural areas of developing nations where impoverished people majorly depend on agriculture and natural resources for their livelihoods [[6], [7], [8]]. However, due to the low profitability associated with livelihoods based on agriculture and natural resources, various studies show that rural areas have shifted from purely agricultural livelihoods to mixed livelihood strategies that are more market-oriented [[9], [10], [11], [12]].

In literature, there are two lines of argument on the poverty-livelihood nexus. The first line of argument focuses on how the poverty status of a household is affected by the household's decision to specialize or diversify their livelihood source(s). Most studies find that when a household's livelihood is specialized on agriculture and allied activities, the household is more prone to poverty than when their livelihood is diversified or specialized on non-agricultural, market-oriented activities [[13], [14], [15]]. The second line of argument focuses on the role of access to different types of livelihood capitals in poverty reduction. Some studies find that access to social capital [16], or physical capital [17], or financial capital [18], or natural capital [19,20], or human capital [21] play a dominant role in poverty reduction. Other studies find that access to more than one of the five capitals is instrumental in the poverty reduction strategy of households [22,23].

In the Indian context, only a few authors have weighed in on both sides of the argument. For example, Mondal et al. [24] analyzed the role of livelihood diversification in abating multidimensional poverty in rural coastal villages of West Bengal. Using the global multidimensional poverty index to estimate poverty and the Simpson's index to estimate livelihood diversification, they found that multidimensional poverty was high, livelihood diversification was reducing, and households were transitioning from agriculture-based livelihoods to other non-farm activities. Similarly, Meher [25] explored the relationship between income poverty and livelihood diversification among tribal populations in Odisha, establishing the impact of the poverty-livelihood nexus on the health of the tribals. The study found that households with higher dependence on agricultural activities were more likely to be poor. Another study [14] used the Indian Human Development Survey data for 2004-05 and 2011-12 to highlight the role of livelihood diversification and social capital in escaping consumption poverty in Eastern India. The study found that non-agricultural livelihood diversification strategies and access to social capital empowered households to escape poverty.

However, there are three major gaps in literature. Firstly, existing studies measure rural poverty through monetary approaches (i.e., the income or consumption approach) or using the global multidimensional poverty index (MPI). While these measures are instrumental in capturing certain aspects of poverty, there is a growing consensus in literature on the ineffectiveness of monetary poverty measures in estimating poverty [26,27] and the inefficiency of macro monetary or multidimensional poverty measures in capturing the specific dimensions of rural poverty [28,29]. Secondly, previous studies establish the relationship between poverty and livelihood by either focusing on livelihood strategies or access to livelihood capitals [24]. By definition, a livelihood system consists of the capabilities, assets, and activities needed to make a living [4]. According to De Haan [30], poor people develop their livelihood strategies based on their access to certain important capitals. This means that a household's livelihood outcome stems from their livelihood strategies, which are determined by their access to livelihood capitals [15]. In other words, the relationship between poverty, which is a livelihood outcome, and access to livelihood capitals is possibly mediated by livelihood strategies. Such a possible mediation has not been tested before in the poverty-livelihood literature. Thirdly, there is a dearth of studies on multidimensional poverty and livelihood in specific rural contexts in India. Micro-level research geared towards understanding livelihood and poverty is essential for assessing the effectiveness of poverty reduction strategies at the macro level [31].

Against this backdrop, we address four objectives using two villages from Bihar, India. The first objective is to measure the extent of rural poverty in the study area using the rural multidimensional poverty index (RMPI) that the Food and Agriculture Organization (FAO) and Oxford Poverty and Human Development Initiative (OPHI) proposed in 2022, thus pioneering its application in the Indian rural context. The second objective is to construct a scalable composite index for measuring access to livelihood capitals using the Alkire-Foster method and to apply it in the study area. The third objective is to assess livelihood diversification in the study area using the Simpson's livelihood diversification index. The fourth objective is to examine the role of livelihood diversification in mediating the relationship between access to livelihood capitals and multidimensional poverty. To address objective four, we specify and test three hypotheses.

1) Rural multidimensional poverty is related to access to livelihood capitals (which is measured as ‘nonaccess to livelihood capitals’ in this study).
2) Nonaccess to livelihood capitals affects livelihood diversification.
3) Livelihood diversification mediates the relationship between livelihood capitals and rural multidimensional poverty.

The current study contributes to the literature by estimating rural poverty with rural-centric measures and evaluating livelihoods from a holistic viewpoint in the context of a developing country (i.e., India), which is relevant for formulating policies and designing intervention programs in rural India and similar geographic regions across the developing world. Theoretically, the study contributes by empirically testing the Sustainable Livelihood Framework's (SLF) proposition that livelihood strategies – measured in terms of livelihood diversification in this study – mediates the relationship between access to livelihood capitals and livelihood outcomes (i.e., multidimensional poverty in this case), thus differentiating from earlier studies in this area [14,24,25].

The rest of the paper is organized as follows. Section two presents the literature review. Section three presents the context, data, and methods. Section four presents the results and discussions. Section five presents the conclusions, policy recommendations, and future research points.

## 2 Literature review

### 2.1 Rurality and poverty

In the literature, two types of definitions of poverty exist: monodimensional and multidimensional. Based on the proponents of monodimensional poverty, poverty refers to the social exclusion and discrimination of certain individuals and households or households' inability to earn/spend according to the stipulated thresholds in the society (i.e., economic lack) or households’ inability to access a proper diet/relevant socio-economic opportunities and facilities [32,33]. Meanwhile, the proponents of multidimensional poverty define poverty as a condition that exposes households to simultaneous deprivations in several aspects of well-being, mainly education, health, and living standards [34].

Considering the argument that poverty is largely a rural, agrarian, and location-centric phenomenon [13,29,35,36] several scholars have proposed that rural poverty should be measured using rural-centric measures [37,38]. This is because the focus of poverty measurement at the global level is comparability, and poverty indicators are chosen based on the similarity and availability of relevant variables in most countrywide databases [39]. To address this issue, several monetary poverty studies used different consumption baskets and poverty lines for rural and urban areas [[40], [41], [42]]. However, in multidimensional poverty studies, this issue has not been addressed. So, in 2022, the Food and Agriculture Organization (FAO) collaborated with the Oxford Poverty and Human Development Initiative (OPHI) to propose a rural-centric measure of multidimensional poverty known as the Rural Multidimensional Poverty Index (RMPI).

The RMPI was formulated to address two conceptual issues in rural multidimensional poverty measurement. The first issue is highlighting some of the specific characteristics of rural poverty to give policymakers room to formulate area-specific pro-poor programs [29]. The second issue is allowing comparability among different livelihood groups (e.g. forest communities, riverine areas, and agrarian communities) across spatial contexts [29]. The RMPI is instrumental in guiding poverty eradication strategies at multiple levels of governance [29]. There are two major differences between the RMPI and the global MPI: 1) the RMPI is made up of two additional dimensions (i.e. rural livelihoods and resources, and risk) that are rural-specific, and 2) the health dimension was renamed food security and nutrition in the RMPI, and the indicators changed from ‘child mortality and malnutrition of any adult or child in the household’ to ‘food insecurity and child malnutrition’ as both indicators are more representative of the rural context [29]. According to FAO and OPHI [29], the reason for.

1. Adding “rural livelihood and resources” as a dimension of rural multidimensional poverty is to pinpoint the contextual livelihood options present in the specific rural area and highlight the resource deprivations hindering households from earning a living from those livelihood options.
2. Including “risk” as a dimension of rural multidimensional poverty is because rural households are exposed to climatic stresses and shocks as they mainly depend on natural resources for survival. Therefore, their level of access to risk coping facilities is an important poverty issue.
3. Replacing “health, which captured child mortality and malnutrition of ANY household member” with “food security and child nutrition” is because food insecurity and child malnutrition are more severe in rural areas than the previous variables specified in the global MPI.

### 2.2 Livelihood mix

According to Chambers and Conway [4], livelihood refers to the “capabilities, assets, and strategies required to make a living”. In the livelihood context, *capabilities* refer to households' ability to cope with livelihood stress (i.e., regular livelihood challenges like illness and unemployment) and shocks (i.e. irregular livelihood challenges like natural disaster), and to access livelihood opportunities [4,43]. Also, *assets* refer to the various resources that households require to make a living, *strategies* refer to the various combinations of economic activities that households can construct to earn a living, and *outcomes* refer to the resources that households gain from participating in several economic activities [4,43]. For a household's livelihood outcome to be progressive and stable, they require a livelihood system that is sustainable [44]. A livelihood is sustainable when: 1) it is resilient to different kinds of stress and shocks; 2) it allows for the improvement of capabilities and assets; 3) it provides livelihood opportunities for the next generation; 4) it contributes net benefits to other livelihoods at multilevel governments over various time periods; and 5) strengthens or protects the natural resource base [4,30,45].

**Fig. 1:**
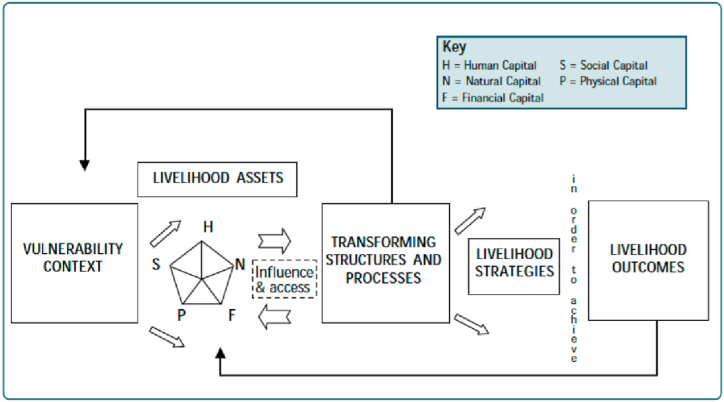
The sustainable livelihood framework.

***Source*:** Stienstra and Lee, 2019.

In literature, the sustainable livelihood framework (SLF), which was proposed by Roberts Chambers in 1983, is the most used livelihood framework for encapsulating the complexities between livelihood inputs and outputs. SLF, as shown in Fig. 1, propounds that “the ability to pursue different strategies depends on the possession of or access to five core assets from which different productive streams are derived and livelihoods are constructed” [46]. The five core assets are natural capital, physical capital, social capital, financial capital, and human capital [4]. Also, there are three broad clusters of livelihood strategies within SLF namely: agricultural intensification/extensification, livelihood diversification, and migration [43]. To offer an operational and simplified description of the combination of livelihood resources/capitals and strategies to yield profitable outcomes, we coin the term *Livelihood Mix*. In other words, *Livelihood Mix* refers to how households’ access to livelihood capitals integrates with their livelihood strategies to result in better livelihood outcomes.

Given that SLF does not present any objective method and algorithm for identifying and quantifying the priority indicators for sustainable livelihoods [44,47], several authors have quantified the livelihood mix through a variety of indicators that mirror the five livelihood capitals and by using several parameters to capture livelihood strategies [48,49]. For instance, studies have estimated livelihood strategies using fuzzy approach [15], simple statistical approaches [14,50,51], Simpson's Livelihood Diversification index [13,24,52,53], Margalef index [54], count index [55] and a combination of indices [56]. However, Simpson's Livelihood Diversification Index (SDI) is the most used because it is robust and easily computed [57].

Also, many authors have adopted several indicators of each of the livelihood capitals using various methods of constructing composite indices like principal component analysis [2], entropy weighting method [58], and several other methods of weighting [59,60]. Nevertheless, there is no consensus in literature on the index to be used for estimating access to livelihood capitals because its indicators are diverse and complex in each geographic area [61]. Therefore, we estimate ‘access to livelihood capitals’ by selecting 18 indicators that succinctly represent the five livelihood capitals of the Sustainable Livelihood Framework through literature review, key informant interviews, and the authors' field experience (see appendix 2). We also reveal the role of government welfare policies (i.e., transforming structures and processes) in extenuating poverty by including indicators that capture awareness and access to government welfare programs under the social capital dimension [43]. This is because relational networks with various actors (i.e., the state, market, and NGOs) in the society aids access to the other four capitals, and creates opportunities and means for turning these four capitals into viable livelihoods [62].

Unlike other studies, we prefer the Alkire-Foster (AF) method of constructing composite indices over other methods like principal component analysis and entropy weighting method because it reflects the joint distribution of deprivations in the different components of the index and yields a single statistic that captures the compound intensity of deprivation in the phenomenon of interest faced by the unit of analysis [27]. Given that the household is mostly the unit of analysis in poverty and livelihood studies [4,63], we adopt the household as our unit of analysis. Although the AF method was originally developed to capture multidimensional poverty [34], several studies have applied it to other socio-economic issues like energy poverty [64], livelihood vulnerability [65], human recognition [66], and women's empowerment [67]. We therefore contribute to literature by adopting it to study access to livelihood capitals.

### 2.3 Nexus between poverty and livelihood

Empirical studies, using both monodimensional and multidimensional poverty measures, establish that poverty relates to different aspects of livelihood. Some studies highlight how lack of access to various livelihood capitals increases poverty [68,69]. Meanwhile other studies find that the adoption of certain kinds of livelihood strategies, especially agriculture-based strategies, leads to high poverty among households [[13], [14], [15]]. Several studies have also explored the livelihood capital-livelihood strategy nexus in poor geographic areas and found that the access to livelihood capitals determine the livelihood strategies of people, thus impacting their socioeconomic outcomes [70,71]. As mentioned in the introduction section, most of the studies on this topic lack in two key aspects: 1) they compare livelihoods with monodimensional poverty measures, and 2) they explore the relationship between poverty and livelihood from the purview of either livelihood strategies/diversification or livelihood capitals. Only a few studies have considered the livelihood-poverty nexus using both aspects of livelihood [72,73], or from the ambit of multidimensional poverty, especially applying the multidimensional poverty index (MPI) [74,75].

## 3 Context, data, and methods

### 3.1 Rationale and locale of the study

Villages are the life of the Indian economy [76] as more than half of India's population reside in rural areas [77]. However, data pinpointing the remoteness dimension of rural areas are often excluded in national databases, especially in developing countries like India, due to the exorbitant cost of data collection [29,78], thus making it very difficult to highlight and rectify the plights of the poor in rural areas. To contribute to addressing rural challenges in India, Mata Amritanandamayi Math, which is a United Nations-recognized international charitable organization, initiated a rural development program called the Amrita Self Reliant Villages (Amrita SeRVe) in 2013 [79,80].

**Fig. 2:**
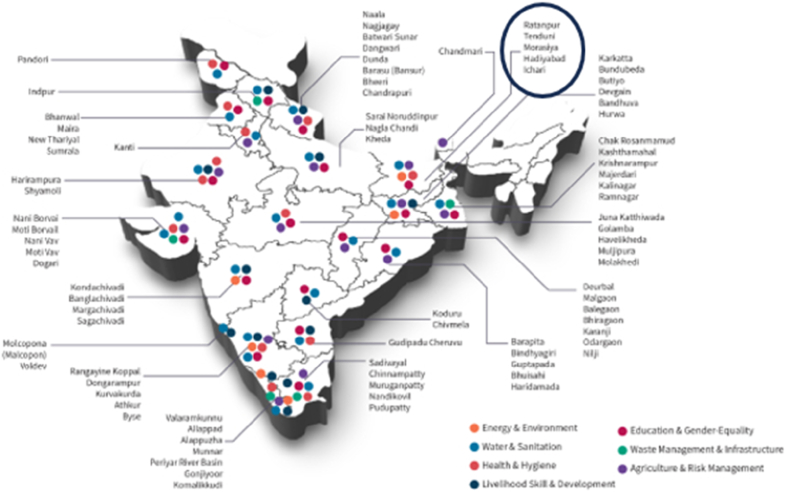
Indian map showing the villages adopted by amrita SeRVe and the Program's reach across various thematic areas of sustainable development.

This program spreads across over 100 villages in 22 Indian states (see Fig. 2). The villages were selected by a team of volunteers that travelled across the 27 states of India and identified the most marginalized communities in backward and isolated village clusters through relevant participatory rural approaches in July 2013 [80]. The reason for this method of village selection is that the organization aimed at identifying the multidimensional pain points of these backward communities, creating solutions for these problems through low-cost-efficient technologies, and scaling these solutions to similarly disadvantaged neighbouring communities [80]. To foster experiential learning among students at the tertiary level, Amrita Vishwa Vidyapeetham University incepted a program called the Live-in-Labs (LiLa), which facilitates multidisciplinary theory-into-practice research in the villages adopted by Amrita SeRVe [79].

***Source:***https://www.amrita.edu/school/sustainable-futures/academics/live-in-labs/.

Of the 22 Indian states included in the Amrita SeRVe and LiLa projects, Bihar is the poorest, least developed, and third most populous Indian state [[81], [82], [83], [84]]. It is a landlocked state and about 88.5 % of its population live in rural areas [85]. Bihar is also home to more than 60 % of historically backward castes [82]. So, we purposively select Bihar as the study's focus. By 2019, Amrita SeRVe and LiLa had adopted five villages in Bihar namely: Ratanpur, Tenduni, Hadiabad, Ichari, and Morasia. Given that we aim to highlight the poverty and livelihood dynamics per village cluster, we purposively select three – Ratanpur, Hadiabad, and Tenduni – out of the five villages for this study because they belonged to the same village cluster while the other two (Ichari and Morasia) belonged to separate village clusters. Of the three villages we select, we exclude Ratanpur because over 75 % of households in the village were beneficiaries of the activities of Amrita SeRVe and Live-in-Labs [86], and our future goal is to scale up our rural development activities to the remaining two villages in the cluster. Therefore, we use both villages as case studies to evaluate the current deprivation status of the village cluster, and to explore how multidimensional poverty is linked to various aspects of livelihood.

**Fig. 3:**
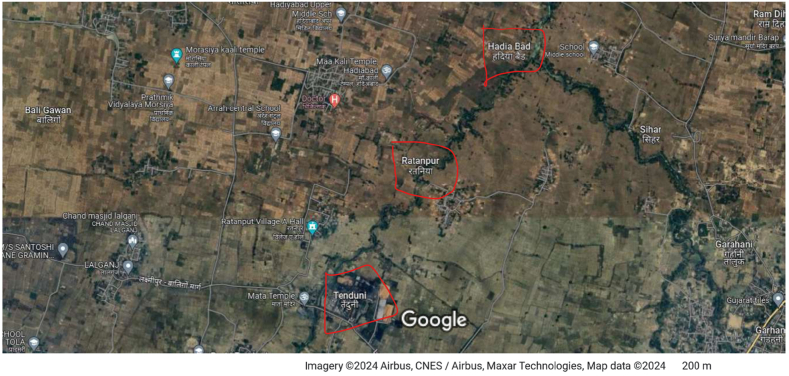
Map of village cluster.

***Source*:** Google map.

Tenduni and Hadiabad are typical agrarian communities located in Garhani Block, Bhojpur District, Bihar, India. We present the map showing the location of both villages in Fig. 3. Given that there is a dearth of village level information at local government data directories, we conducted four Key Informant Interviews (KIIs) with community leaders and spokespersons, as well as present/past local government leaders – also known as Panchayat Head or *Mukhiya* – to obtain the socio-demographic information for the villages. The KIIs revealed that the main occupation in both villages is crop and livestock farming. However, structural transitioning to non-agricultural livelihood activities has occurred in both villages due to less rainfall, lack of access to irrigation facilities [82], and the invasion of Nilgai [87]. So, most agricultural activities in both villages are done at subsistence levels. In Tenduni village, there were 70 households and more than half (52.4 %) belonged to the scheduled caste (SC) while in Hadiabad there were 160 households and 15 % of them were Muslims. Also, about 53 % and 67 % of households in Hadiabad and Tenduni, respectively, were landless. Both villages are interesting contexts for poverty analysis because they are agrarian yet more than half of the villagers were landless and each village comprised of a historically impoverished subgroup – that is, Muslims and SCs – in India [88].

### 3.2 Data collection techniques

The present study relies on primary data collected using a structured questionnaire. Through household surveys, we obtained information on the poverty-livelihood status of households. Also, we conducted eight key informant interviews (KII) to gather some descriptive information about the villages to support our quantitative analysis. The first four KIIs, as stated in section 3.1, were to obtain the socio-demographic information for the villages. Meanwhile the last four KIIs, which were conducted with government officials from relevant state departments, were to find out the government schemes for different categories of people in the village and how the villagers can access the required schemes to enhance their livelihoods. Field work was conducted from December 2023 to February 2024.

For Tenduni village, we adopt census method for the household interviews since the village comprises of 70 households, which is a very small number. Out of 70 households, only 63 consented to participate in the study, leading to a mortality of 7 households. Of the 7 households, 2 were excluded because they currently reside outside the community, 1 household refused participation, and 4 households were excluded after three visits either because their doors were locked, or no eligible adult was present to consent to the study. Seeing that some households in Hadiabad were unresponsive, we adopt judgemental sampling techniques where the village coordinator of Hadiabad (i.e. a key informant) identified households that will likely be willing to participate in the study. We enlisted about 105 households, of which 81 participated in the study while 24 were either unavailable to respond or did not consent to the study. Of the 81 households interviewed, 1 household was dropped due to missing information. In total, 143 households participated in this study from both villages.

### 3.3 Measuring rural multidimensional poverty

The rural multidimensional poverty index (RMPI) measures the overlapping deprivations that poor people face in rural areas by accounting for the rural economy and its contributions to the living conditions of rural people [29]. It comprises of five dimensions and eighteen indicators (see Appendix 1) that are equally weighted [29]. A rural household is classified as multidimensionally poor if it is deprived in 33.3 % of the weighted indicators [29]. The Alkire-Foster [89] methodology identifies the poor using two cutoffs namely: the deprivation cutoff and the poverty cutoff [34]. Under the deprivation cutoff or first order cutoff, a household is classified as deprived in an indicator if they fall short of the benchmark set for the indicator (see Appendix 1). Under the poverty cutoff or second order cutoff, a household is poor if they are deprived in one-third of the weighted indicators [29].

RMPI is the product of two partial indices: the headcount ratio (H) and the poverty intensity (A). Mathematically, the RMPI can be denoted by:

RMPI=H×A

Where H is denoted by:

$$H=\frac{n^{p}}{n^{O}}$$

Where $n^{p}$ represents the number of poor people in the population and $n^{O}$ gives the number of people in the population. The quotient of $n^{p}$ and $n^{O}$, otherwise known as *H*, is the proportion of poor people in the population.

Where, A is denoted by:

$$A=\sum_{j=1}^{N^{p}}w_{j}d_{j}$$

Where $N^{p}$ is the number of poor households, $w_{j}=\frac{n_{j}}{n^{p}}$ is the share of the jth household in the total number of poor, $n_{j}$ is the number of persons in household ‘j’, $n^{p}$ is the amount of poor people in the population, $d_{j}$ is the number of dimensions in which household ‘j’ is impoverished. The entire equation reveals the average number of weighted deprivations faced by poor households in the population. For more details on the deprivation cutoffs and weight distribution among indicators, see Appendix 1.

### 3.4 Sustainable livelihood framework (SLF)

**Fig. 4:**
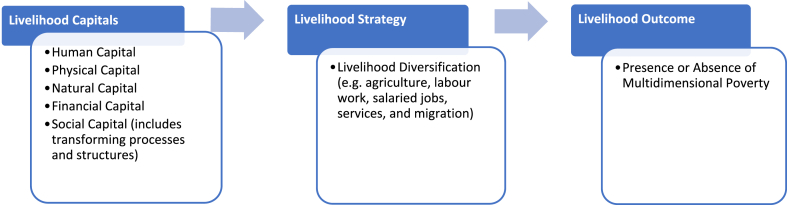
Conceptual Framework Based on SLF.

***Source*:** Modified from DFID's sustainable livelihood framework as presented in Krantz [43].

In the current study, we use SLF as the theoretical backdrop to explain the *Livelihood Mix* of households. In Fig. 4, we present our conceptual framework, which is an adaptation of our study's objectives to the Sustainable Livelihood Framework. Our model prioritises: 1) ‘the composite access to livelihood capitals’ as the main variable under livelihood capitals, 2) livelihood diversification, as the main variable under livelihood strategies, and 3) poverty as the variable for livelihood outcome. To estimate livelihood diversification and multidimensional poverty, we employ Simpson's diversification index and rural multidimensional poverty index, respectively. To capture the access to livelihood capital, this paper creates a composite livelihood capital index using the Alkire-Foster methodology.

#### 3.4.1 Simpson's livelihood diversity index (SDI)

Due to its simplicity, robustness, and wide applicability, the Simpson's livelihood diversity index (SDI) is the most widely used measure of livelihood diversification and vulnerability in literature [57,90]. According to Swargiary and Mahanta [91], SDI is denoted by:

$$SDI=1-\sum_{i=1}^{N}P_{i}^{2}$$

N is the total number of livelihood sources available to households in the study area and $P_{i}$ is the proportion of income that a household accrues from livelihood source ‘*i*’. The value of the index varies from 0 to 1 [92]. When the value of livelihood diversification for a household is zero (0), it implies that the household is dependent on one livelihood source. But the value tends to one as the household's streams of income increases [93]. According to Mondal et al. [24], there are five classes of household livelihood diversification based on SDI values. They are:

• No diversification (if SDI is less than 0.1)
• Low diversification (if SDI is between 0.1 and 0.25)
• Medium diversification (if SDI is between 0.26 and 0.50)
• High diversification (if SDI is between 0.51 and 0.75)
• Very High diversification (if SDI is above 0.75)

#### 3.4.2 Nonaccess to livelihood capital index (NLCI)

To address the first part of objective 2, which is to construct a composite index for measuring access to livelihood capitals, the current study leverages results from key informant interviews, field experience, and literature review. Using these techniques, we identify the indicators for capturing access to livelihood capitals and set the livelihood deprivation cutoffs, which we present in appendix 2. To capture the composite and simultaneous access to livelihood capital, we employ the Alkire-Foster (AF) methodology, which identifies the lack of composite access to livelihood capitals using two cutoffs namely: the first order cutoff and the second order cutoff [34]. Under the first order cutoff, a household is classified as deprived in any livelihood indicator if they fall short of the benchmark set for the indicator (see Appendix 2). Under the second order cutoff, a household is deprived in composite access to livelihood capitals if they are deprived in one-third of the weighted livelihood indicators [29].

NLCI is the product of two partial indices: the headcount ratio (H) and the intensity of the non-composite access to livelihood capitals (A). Mathematically, the NLCI can be denoted by:

NLCI=H×A

Where H is denoted by:

$$H=\frac{n^{p}}{n^{O}}$$

Where $n^{p}$ represents the number of people in the population that lack composite access to livelihood capitals and $n^{O}$ gives the number of people in the population. The quotient of $n^{p}$ and $n^{O}$, otherwise known as *H*, is the proportion of people in the population that cannot compositely access livelihood capitals.

Where, A is denoted by:

$$A=\sum_{j=1}^{N^{p}}w_{j}d_{j}$$

Where $N^{p}$ is the number of households that cannot compositely access livelihood capitals, $w_{j}=\frac{n_{j}}{n^{p}}$ is the share of the jth household in the total number of people that do not have composite access to livelihood capitals, $n_{j}$ is the number of persons in household ‘j’, $n^{p}$ is the amount of people in the population that cannot compositely access livelihood capitals, $d_{j}$ is the number of livelihood dimensions in which household ‘j’ is deprived. The entire equation reveals the average number of weighted deprivations faced by households in the population that cannot compositely access livelihood capitals. For more details on the deprivation cutoffs and weight distribution among indicators, see Appendix 2.

The values of the NLCI, as prescribed by the AF methodology, ranges from zero to one. The closer the household deprivation score to zero, the higher their access to livelihood capitals, and the closer the household deprivation score to one, the higher their nonaccess to livelihood capitals. Given that the current study focuses on highlighting the challenges associated with nonaccess to livelihood capitals, we describe access to livelihood capitals in terms of nonaccess in the succeeding sections of the paper.

### 3.5 Estimation technique

To test hypotheses 1 to 3, we adopt the Baron and Kenny [94] approach as it is the most popular method for capturing mediation effects and to test mediation models that stem from theories [95]. This approach tests mediation effects using a linear regression process that must satisfy two conditions through three regression models [94,95]. Linear regression modelling, as prescribed by the adopted mediation approach, is suitable for the present study because the linear regression model used for this analysis emerged as the best model, in terms of the goodness of fit, when compared to the non-linear models. We run the regression models using the IBM SPSS Software (version 26).

The first regression model, as shown in equation (1), gives the total effect ($\beta_{1}$) of the independent variable – Nonaccess to Livelihood Capital Index (NLCI) – on the dependent variable – Rural Multidimensional Poverty Index (RMPI), thus testing hypothesis 1.

(equation 1) $$RMPI=\beta_{0}+\beta_{1}NLCI+e_{i}$$

The second regression model, as shown in equation (2) below, gives the first component of the indirect effect ($\beta_{2}$) by examining the effect of the independent variable (i.e., NLCI) on the mediating variable (i.e., Simpson's Livelihood Diversification Index – SDI), thus testing hypothesis 2.

(equation 2) $$SDI=\beta_{0}+\beta_{2}NLCI+e_{i}$$

The third regression model, as shown in equation (3), gives the second part of the indirect effect ($\beta_{3}$), and the direct effect ($\beta_{4}$), which depict the effects of the mediating variable (i.e., SDI) and the independent variable (i.e., NLCI), respectively, on the dependent variable (i.e., RMPI), and test hypothesis 3.

(equation 3) $$RMPI=\beta_{0}+\beta_{4}NLCI+\beta_{3}SDI+e_{i}$$

We can conclude that SDI completely mediates the nexus between RMPI and NLCI only if the two conditions below are satisfied.

1) $\beta_{1}\text{,}$$\beta_{2}\text{,}$ and $\beta_{3}$ are statistically significant.
2) $\beta_{4}$ is less than $\beta_{1}$.

Nonetheless, if the first condition is met and the second condition is unmet, we can conclude that SDI partially mediates the nexus between RMPI and NLCI. Any other results derived imply that mediation is absent in the model.

To capture the strength of the mediation effect, we use the proportion test shown in equation (4). The proportion test gives the percentage of the total indirect effect (i.e., the product of $\beta_{2}$ and $\beta_{3}$) to the total effect (i.e., $\beta_{1}$) of the models.

(equation 4) $$Proportion\;Test=\frac{Indirect\;Effect}{Total\;Effect}\times 100=\frac{\beta_{2}\times \beta_{3}}{\beta_{1}}\times 100$$

To test the significance of the mediation effect, we run the Sobel's [96] test using the formulae given in equation 5

(equation 5) $$Z\;Value=\frac{a\times b}{\sqrt{\left(b^{2}\times s_{a}^{2}\right)+\left(a^{2}\times s_{b}^{2}\right)}}$$

**Fig. 5:**
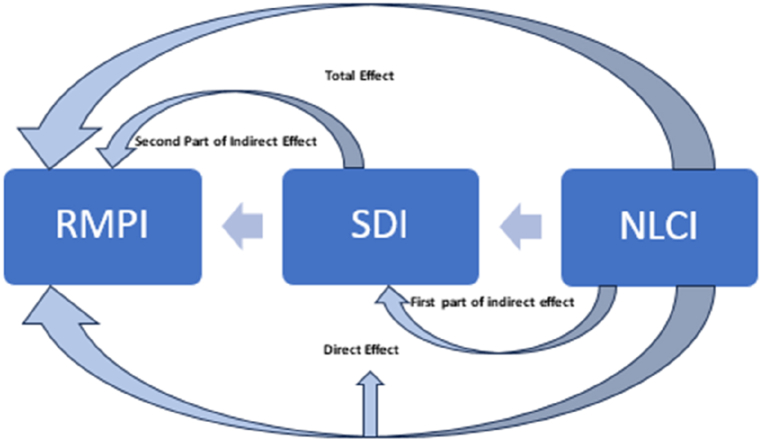
Relationship between variables of interest.

***Source*:** Authors' visualization of regression model.

Where a is the unstandardized coefficient of the independent variable, $s_{a}$ is the standard error of the independent variable, b is the unstandardized coefficient of the mediation variable, and $s_{b}$ is the standard error of the mediation variable. Fig. 5 gives a pictorial summary of our model.

## 4 Results and discussions

### 4.1 Descriptive statistics

Before we present the estimation results, we present the summary statistics of the household demographics in both villages (see Table 1). The figures show that the average age of the household head in both villages is 56 years. Around 88 % of the household heads in both villages were male and the rest were females. Also, 60 % of household heads from both villages were educated at least up to primary level. In terms of caste, about 30 % and 36 % of household heads were from general category (GC) and other backward castes (OBC) respectively. Meanwhile, 26 % of household heads from both villages belonged to the scheduled castes (SC) and 8 % were casteless (NC). The average household size in both villages was about eight with an average of five adults and three children in each household. In terms of religion, about 90.2 % of households in both villages were Hindu while 9.8 % were either Muslim or Christian. Also, about 59 % of households in both villages were landless (i.e. they did not own agricultural land) with Tenduni recording 67 % of landless households and Hadiabad recording 53 %. The average income of each household head in both villages was Rs. 4361 per month.

**Table 1:** Descriptive Statistics of the Demographics of the villages.

S/No	Indicators	Statistics	Group	Tenduni (N = 63)	Hadiabad (N = 80)	Both (N = 143)
1	Age of Household Head (in years)	Mean	–	54	58	56
		Standard Deviation	–	16.6	13.7	15
		Range	–	24–100	30–80	24–100
2	Income of Household Head (in rupees per month)	Mean	–	4398	4331	4361
		Standard Deviation	–	5358	5493	5415
		Range	–	0–30,000	0–20,000	0–30,000
3	De Facto Household Size (in Numbers)	Mean	–	8	8	8
		Standard Deviation	–	5	4	4
		Range	–	2–35	2–19	2–35
4	Number of Adults per household	Mean	–	4	5	5
		Standard Deviation	–	3	2	3
		Range	–	2–17	2–13	2–17
5	Number of Children per household	Mean	–	3	3	3
		Standard Deviation	–	3	2	2
		Range	–	0–18	0–7	0–18
6	Education of Household Head	Frequency (%)	No Education	41.3	38.8	40
			Primary	17.5	13.8	15
			Middle	12.7	7.5	10
			Secondary	20.6	33.8	28
			Higher Secondary	3.2	1.3	2
			Graduation	0	3.8	2
			Post Graduation	4.8	1.3	3
7	Gender of Household Head	Frequency (%)	Males	92.1	85	88
			Females	7.9	15	12
8	Household's Caste	Frequency(%)	General Category (GC)	28.6	31.3	30
			Other Backward Caste (OBC)	19	48.8	36
			Scheduled Castes (SC)	52.4	5	26
			No Caste (NC)	0	15	8
9	Religion	Frequency(%)	Hindu	96.8	85	90.2
			Muslim	0	15	8.4
			Christian	3.2	0	1.4
10	Land Ownership	Frequency (%)	Landowner	33.3	47.5	41
			Landless	66.7	52.5	59

### 4.2 RMPI, NLCI, and SDI in the chosen villages

To address objectives 1 to 3, Table 2 presents the values of RMPI and its components, NLCI and its components, and SDI for the chosen villages. The estimates of RMPI revealed that 4.2 % of people are deprived in an average of 35 % of the weighted indicators. However, we observe that households from both villages were most deprived in extension services (100 %), followed by food security (59.4 %), sanitation (46.9 %), assets (36.4 %), and cooking fuel (34 %). According to the information obtained from the KIIs, the low RMPI in our study area could be attributed to overdependence on government schemes. The key informants reveal that in the dimensions of poverty where the communities faced the least deprivations, the coverage of the relevant government scheme was high. Meanwhile, for most aspects of poverty where deprivation rates were high (i.e., extension services, food security, sanitation, assets, and cooking fuel), the communities were either unaware of the relevant government scheme or unsatisfied with the implementation and delivery of the relevant scheme so they used it less. For example, most of the beneficiaries of the public distribution system, which provides rural households with food grains, reported that they do not use that service because the quality of food distributed is substandard and others reported that some of their family members’ names were removed from the ration card (i.e., the pass for accessing the scheme), thus implying that the quantity of food received was not at par with their family size. Using information from the household interviews, we support the findings from the KIIs by comparing the incidence of deprivation per indicator to the percentage of beneficiaries for the relevant government schemes in Table 3. Evidence from Table 3 further validates the opinions of the key informants that the low RMPI in the study area does not equate to improved economic wellbeing in the community because the community continues to be on state support for basic essentials.

**Table 2:** RMPI, NLCI, and SDI for the chosen villages.

Village	Rural Multidimensional Poverty (RMPI)			Nonaccess to Livelihood Capitals (NLCI)			Simpson's Livelihood Diversification Index (SDI)
	*H*	*A*	*RMPI*	*H*	*A*	*NLCI*	*SDI*
Tenduni	0.048	0.374	0.018	0.777	0.485	0.377	0.098
Hadiabad	0.037	0.339	0.013	0.810	0.512	0.415	0.153
Both	0.042	0.357	0.015	0.795	0.560	0.445	0.129

**Note:** H stands for Headcount ratio and A stands for Intensity of poverty/Non access to livelihood capitals.

**Table 3:** Matching RMPI indicators with relevant government schemes.

RMPI Indicator	Incidence of Deprivation (in %)	Relevant Government Scheme/Facility	Coverage (in %)	References for full details on the relevant schemes
Food Insecurity	59.4	Ration cards to access the public distribution system (PDS)	100	[97]
Child Malnutrition	17.5	Midday meal scheme operational in government-sponsored nursery schools or *Anganwadi* and Tuition free government schools (up till junior secondary level or standard 10).	100	[98]
Years of Schooling	3.5			
School Attendance	0.7			
Child Labour	0			
Cooking Fuel	34	Pradhan Mantri Ujjwala Yojana	66	[99]
Sanitation	46.9	Swachh Bharat Mission	54.5	[100]
Drinking Water	0	Mukhyamantri Gramin Peyjal Nishchay Yojana	100	[101]
Electricity	0	Saubhagya scheme	100	[102]
Housing	13.3	Pradhan Mantri Awas Yojana	86.7	[103]
Assets	36.4	No scheme	–	–
Low Pay Rate	2.1	MGNREGA	18.9	[104]
Social Protection	2.8	Pension Scheme	97.2	[105]
Extension Services	100	Krishi Vigyan Kendra	0	[106]
Agricultural Assets Adequacy	0.7	1) PM Kisan	2.1	[107]
		2) Jeevika scheme	100	[108]
Credit Denial	0	Jeevika scheme	100	[108]
Risk Exposure and Coping Strategies	0			

Table 2 also reveals that nonaccess to livelihood capitals is a pertinent issue in the study area as 79.5 % of households were deprived in an average of 56 % of the weighted indicators. When we decompose the NLCI by indicator, we find that all (100 %) households in both villages were deprived in savings, revealing that households in both communities are vulnerable to multifaceted livelihood and environmental shocks. The second highest deprivation in access to livelihood capital stemmed from women's unemployment (93.7 %). This finding corroborates that of Kanyagui et al. [109] in rural Uttar Pradesh. According to Deepika and Sigi [110], unemployment, especially among women, is a general livelihood issue in India. Several studies [111,112] have identified women's participation in the labour force as paramount for poverty reduction in rural areas. So, a high deprivation in women's employment in both communities reveal that households are exposed to precarious livelihoods and poverty. This is also validated by our findings from the KIIs, which reveal that widows and their children in both communities fall into dire economic situations immediately after their spouse, who is the breadwinner, dies because they are both unemployed and unskilled. Other sources of high deprivation in access to livelihood capitals in the study area include poor income (88.8 %), lack of skill training (86 %), and landlessness (58.7 %). Several studies also show that access to better income [113], skill training [111], and agricultural land [114] play significant role in poverty reduction in rural areas. Lastly, households in both villages were least deprived in neighbourhood relations (1.4 %), and health (4.2 %).

Furthermore, Table 2 reveals that the combined SDI value for both villages was 0.129, which means that there is low livelihood diversification in both villages. The SDI for Tenduni (0.098) shows no diversification while that of Hadiabad (0.153) shows low diversification. From Table 4, we see that 58 % of household heads were gainfully employed while the rest were either homemakers or unemployed. We further find that the dominant livelihood strategy was non-farm strategies as 64 % of employed household heads engaged in non-agricultural sources of livelihood. This finding highlights that structural transitioning from agriculture to other sectors have occurred in our study area, as is common in the modern-day rural economies [15,92]. Eleven livelihood activities happen in both villages namely: farming, agriculture labour, construction labour, a mix of both agriculture and construction labour, factory labour, other labour services like house painting and carpentry, business, salaried jobs and services, animal husbandry, government job, and migration. Of these activities, most of the household heads in both villages (23.1 %) primarily involve in construction work (see Table 4). The livelihood diversification in both villages occurs when the household head and other eligible members of the household engage in multiple livelihood activities. It was only in one Hadiabad household that the household head was the only eligible adult and he had two sources of income namely: farming and a salaried job.

**Table 4:** Primary occupation of household head.

Occupation	Both Villages		Hadiabad		Tenduni	
	Frequency	Percent	Frequency	Percent	Frequency	Percent
Farming	15	10.5	10	12.5	5	7.9
Agriculture Labour	12	8.4	8	10.0	4	6.3
Construction Labour	33	23.1	12	15	21	33.3
Agriculture and Construction Labour	1	0.7	0	0	1	1.6
Factory Labour	4	2.8	3	3.8	1	1.6
Other Labour Services	1	0.7	1	1.3	0	0
Business	5	3.5	4	5.0	1	1.6
Salaried Jobs and Services	6	4.2	3	3.8	3	4.8
Animal Husbandry	2	1.4	0	0	2	3.2
Government Job	1	0.7	0	0	1	1.6
Migration	3	2.1	3	3.8	0	0
Homemaker	11	7.7	6	7.5	5	7.9
No Job	49	34.3	30	37.5	19	30.2
Total	143	100.0	80	100.0	63	100.0

In spite of the low diversification and high nonaccess to capital in the study area, the results from the KIIs and household interviews show that rural multidimensional poverty was low because most households in the chosen villages were simultaneously benefitting from most of the relevant government schemes. Therefore, it is not due to the improved socioeconomic capabilities of the villagers that the RMPI was low. It is because most of the relevant facilities were government-provisioned, and despite the glitches in the implementation of some schemes, they provided a good amount of benefits to many households. While the role of government welfare schemes in poverty alleviation cannot be undermined, overdependence on government schemes is counterproductive for poverty reduction because it prevents households and communities from becoming self-reliant, and promotes learned helplessness [115,116]. This fact is already evident in the study area as the communities have failed to improve their wellbeing and livelihoods because they depend on government welfare schemes for survival. For example, some households, who were past beneficiaries of the sanitation scheme, reverted to open defecation when their sanitary facility got damaged because they expected the government to both provide and maintain the facility, thus remaking them deprived in toilet ownership. Therefore, there is a need for the government and relevant stakeholders to formulate policies and design intervention programs that will improve households’ access to livelihood capitals, encourage livelihood diversification, and empower both communities to become self-reliant, thus sustaining rural poverty reduction.

### 4.3 Relationship between RMPI and composite access to livelihood capitals through livelihood diversification

In line with the study's fourth objective, we present the regression results obtained from running the models specified with equations (equation 1), (equation 2), (equation 3)) presented in section 3.5 of the paper. From Table 5, Table 6, Table 7 presented below, it is evident that the R-square values of our regression models are low, which could be due to the small cross-sectional sample size and the location-specific nature of the variables employed [117]. However, models with low R-square values are interpretable if most of the independent variables included in the model have a significant relationship with the dependent variable [118]. Since the study's results satisfy this criteria, we proceed with interpreting the models.

**Table 5:** Model 1 – total effect.

Regression Summary					
R		R Square	Adjusted R Square	Standard Error	
0.468		0.219	0.214	0.076	
**Coefficients**					
	Unstandardized Coefficients		Standardized Coefficients		
	β	Standard Error	Beta	T	Sig.
(Constant)	0.016	0.025	–	0.662	0.509
NLCI	0.302 ($\beta_{1}$)	0.048	0.468	6.291	0.000

Dependent Variable – Rural Multidimensional Poverty Index (RMPI); Independent Variable – Nonaccess to Livelihood Capital Index (NLCI).

**Table 6:** Model 2 – first component of indirect effect.

Regression Summary					
R		R Square	Adjusted R Square	Standard Error	
0.302		0.094	0.088	0.194	
**Coefficients of the Model**					
	Unstandardized Coefficients		Standardized Coefficients		
	β	Standard Error	Beta	t	Sig.
(Constant)	0.361	0.063	–	5.743	0.000
NLCI	−0.470 ($\beta_{2}$)	0.123	−0.307	−3.825	0.000

Dependent Variable – Simpson's Livelihood Diversification Index (SDI); Independent Variable - Nonaccess to Livelihood Capital Index (NLCI).

**Table 7:** Model 3 – direct effect and second component of indirect effect.

Regression Summary							
R		R Square		Adjusted R Square		Standard Error	
0.482		0.232		0.221		0.075	
**Coefficients of the Model**							
	Unstandardized Coefficients		Standardized Coefficients			Collinearity Statistics	
	β	Standard Error	Beta	t	Sig.	Tolerance	VIF
(Constant)	0.35	0.027	–	1.276	0.204		
NLCI	0.278 ($\beta_{4}$)	0.050	0.431	5.541	0.000	0.906	1.104
SDI	−0.051 ($\beta_{3}$)	0.033	−0.121	−1.556	0.122	0.906	1.104

Dependent Variable - Rural Multidimensional Poverty Index (RMPI); Independent Variables - Nonaccess to Livelihood Capital Index (NLCI) and Simpson's Livelihood Diversification Index (SDI).

From the results of Model 1 shown in Tables 5 and it is evident that $\beta_{1}$ (0.302) is statistically significant, thus confirming Hypothesis 1 and implying that Rural multidimensional poverty index has a positive and significant relationship with nonaccess to livelihood capital index. This means that households with higher inabilities to access livelihood capitals had higher poverty deprivation scores. Our finding also supports that of a few other studies, which found that a household's ability to access and effectively utilize livelihood capitals enables them to build resilient livelihoods, thus shielding them from poverty [119,120]. Therefore, access to livelihood capitals has a significant role in reducing rural poverty.

From Model 2, we observe that $\beta_{2}$ (−0.470) is statistically significant, thus confirming Hypothesis 2 and implying that nonaccess to livelihood capitals has a negative and significant relationship with livelihood diversification (see Table 6 below). This means that households with higher inabilities to access livelihood capitals have lesser opportunities to diversify their livelihoods. This finding corroborates that of various studies, which find that households’ inability to access livelihood capitals stalls their ability to diversify their livelihoods [121,122]. So, it is important for households to have access to the required capitals that enables them to derive an effective livelihood mix, which will catalyze the formulation of profitable livelihood strategies, and improve their livelihood outcomes in return [122].

Model 3, as shown in Table 7, reveals that $\beta_{3}$ (−0.051) is not statistically significant. The results show that livelihood diversification has a negative relationship with rural multidimensional poverty but the relation is not statistically significant. Given that $\beta_{1}$ and $\beta_{2}$ are statistically significant but $\beta_{3}$ is not, the model fails to satisfy the first mediation condition (wherein $\beta_{1}\text{,}$ $\beta_{2}\text{,}$ and $\beta_{3}$ should be statistically significant). However, seeing that $\beta_{4}$ (0.278) is less than $\beta_{1}$ (0.302), the model satisfies the second mediation condition (i.e., $\beta_{4}$ is less than $\beta_{2}$). Since the models only satisfy the second mediation condition, we can conclude that mediation is not present. This finding is also supported by the proportion test, whose value (i.e., 7.91 %) shows that the strength of SDI's mediation in the relationship between RMPI and NLCI is very small and Sobel's [96] test, which reveals that the mediation effect of the model is insignificant. Therefore, we reject Hypothesis 3, thus establishing that SDI does not mediate the nexus between RMPI and NLCI as suggested by the sustainable livelihood framework (SLF).

This means that, even if a household is not capable to effectively wield their livelihood capitals to formulate a profitable livelihood strategy, their livelihood outcomes will be positive if their composite access to various capitals is high. For example, in terms of natural capital, our KIIs and household interviews reveal that land-owning households are unable to commercially utilize this resource due to lack of knowledge of modern agriculture practices, lack of irrigation facilities, and Nilgai invasion. But despite being unable to convert this resource into a profitable livelihood strategy, they use it for subsistence farming, thus yielding low food insecurity among land-owning households, which is a livelihood outcome. The same thing applies to livestock ownership, which is a physical capital. Households owning livestock use it for meeting their meat and dairy needs; they only sell the surplus. Although these households are unable to effectively use their access to physical capital to yield entrepreneurship in livestock related businesses, they obtain some livelihood benefits from owning livestock. These findings reiterate the importance of accessing livelihood inputs in improving livelihood outputs [23].

From Tables 7 and it is also evident that the value of VIF (i.e., 1.104) is below 5 and the tolerance (i.e., 0.906) is above 0.25, thus indicating that multicollinearity is moderate in the model and the interpretations of the relationships in the model are tenable.

## 5 Conclusions, policy recommendations, and future research

This paper investigated the levels of rural multidimensional poverty, access to livelihood capitals, and livelihood diversification in two rural communities in Bihar, India. It also empirically tested the SLF proposition that livelihood strategies (i.e., livelihood diversification) mediates the relationship between access to livelihood capitals and livelihood outcomes (i.e., rural multidimensional poverty) in the rural, Indian context. The study has four key findings. Firstly, using the RMPI, which is a new rural poverty measure, we found that rural multidimensional poverty was low in the study area. According to the key informants, the low poverty levels in the communities stem from households’ overdependence on government schemes. Also, deprivations in extension services, food security, sanitation, assets, and cooking fuel were the most severe in our study area. Secondly, from the SDI measured to analyze livelihood diversification, we find that diversification was low in both the villages and the main occupation of the households was construction work, indicating that the community has transitioned from dependence on agricultural livelihoods to non-agricultural livelihood sources.

Thirdly, the present study used literature review, key informant interviews, and field experience to construct a livelihood capital index comprising of eighteen indicators that measures the level of nonaccess to livelihood capitals (i.e. the NLCI) in a given context. The study aggregated the composite index using the Alkire-Foster method because it shows the deprivations people face at the same time. The index measured revealed that there was a high level of nonaccess to livelihood capitals in the chosen communities. The NLCI further revealed that deprivations in savings, unemployment, poor income, lack of skill training, and landlessness were the highest livelihood deprivations in our study area. Fourthly, we find that livelihood diversification does not mediate the relationship between rural multidimensional poverty and access to livelihood capitals. So, we conclude that access to livelihood capitals is more important for poverty eradication than livelihood diversification. This finding challenges SLF's proposition that a household's livelihood outcome depends on their livelihood strategies, which depends on their access to livelihood capitals.

Therefore, we recommend that the Indian government's welfare schemes focus on enhancing access to livelihood capitals for the poor population. Such schemes might equip them to become self-reliant, thus helping to reduce multidimensional poverty. In the areas of poverty and livelihoods where deprivation is severe, we suggest that the government addresses the bottlenecks in the relevant government schemes such that it reduces the abovementioned deprivations and empowers poor people to become self reliant. Also, we propose that the relevant government departments and agencies should invest in creating awareness about government initiatives that drive self-sufficiency in rural areas. Such awareness programs will enable rural dwellers to access better opportunities to construct a profitable livelihood mix, thus improving their socioeconomic well-being.

Finally, the current study has two limitations. First, we observe from our field work that, although the RMPI is efficacious in representing rural poverty, it does not encapsulate all the relevant aspects of rural poverty. In the study area, which might be the case in other rural areas across the globe, overdependence on government schemes seems to be an important dimension of poverty that was excluded from the RMPI. This gives room for future studies to critically appraise the RMPI and include relevant dimensions of rural poverty that are missing in the index. Second, this study is based on cross-sectional and location specific data. So, it fails to capture the trends in poverty-livelihood nexus in both communities, and its findings cannot be generalized, even though it can serve as evidence to corroborate findings in similar rural geographic areas and to inform broad-based rural development policies.

## CRediT authorship contribution statement

**Ogbonna Amarachi Onyeyirichi:** Writing – review & editing, Writing – original draft, Methodology, Funding acquisition, Formal analysis, Data curation, Conceptualization. **M.G. Deepika:** Supervision, Methodology, Formal analysis, Conceptualization.

## Ethical approval

Ethical approval for this study was granted by the Institutional Human Ethics Committee (IHEC) of Amrita School for Sustainable Futures in November 2023. We explained the pros and cons of this study to all participants and obtained a signed informed consent certificate from all human participants of this study. Throughout the study, we strictly adhered to the ethical guidelines and ensured that the identity of all participants was always protected.

## Data availability

The data presented in this study are available on request from the corresponding author.

## Declaration of competing interest

The authors declare that they have no known competing financial interests or personal relationships that could have appeared to influence the work reported in this paper.

## Figures and Tables

**Table:** 

Dimensions	Weights (per dimension)	Indicators	Weights (per indicator)	Deprivation Cut-Off (Deprived if …)
Food Security and Nutrition	1/5	Food Insecurity	1/10	The household's probability of being severely food insecure exceeds 50 percent.
		Child Malnutrition	1/10	At least one child (aged 6–60 months) of the household is underweight and/or stunted.
Education	1/5	Years of Schooling	1/10	No household member of ages “school entrance age +6” or more has completed six years of schooling.
		School Attendance	1/10	Any school-aged child is not attending school up to the age at which he/she would complete class 8.
Living Standards	1/5	Cooking Fuel	1/30	A household cooks with dung, agricultural crops, shrubs, wood, charcoal, or coal.
		Sanitation	1/30	The household has unimproved or no sanitation facility or it is improved but shared with other households.
		Drinking Water	1/30	The household does not have access to improved drinking water or safe drinking water is at least a 30-min walk from home (as a round trip).
		Electricity	1/30	The household has no electricity.
		Housing	1/30	The household has inadequate housing: the floor is made of natural materials, or the roof or wall are made of rudimentary materials.
		Assets	1/30	The household does not own more than one of these small assets: radio, TV, telephone, computer, animal cart, bicycle, motorbike, or refrigerator; and does not own any big asset such as car or truck.
Rural Livelihoods and Resources	1/5	Agricultural Assets Adequacy	1/25	The household's share of income from agriculture (excluding agricultural wages) is equal to or above 30 % and the amount of either land or livestock owned or operated falls in the bottom 40 % of the cumulative distribution of operated land size (ha)/livestock ownership (TLU).
		Low Pay Rate	1/25	At least one household member is a low-paid employee in either (a) agriculture, (b) mining, quarrying, manufacturing, or construction, (c) services, or (d) any other unspecified sector.
		Social Protection	1/25	No member of the household has enrolled in any pension, insurance, or other social programme.
		Child Labour	1/25	At least one household member under the age of 11 years is employed.
		Extension Services	1/25	No one in the household has access to any extension service.
Risk	1/5	Credit Denial	0.75/15	If the household was turned down in all its attempts to seek credit, or the household did not borrow because it did not seek credit due to non-adequate reasons, such as (a) believing the credit would be refused, (b) the credit was too expensive, (c) inadequate collateral, or (d) did not know any lenders.
		Risk Exposure and Coping Strategies	1.125/15	The household suffered from covariate shocks or suffered from a shock but had no access to formalized coping strategies such as support from the government or a non-governmental organization.
		Risk of Climate Shocks	1.125/15	The household's probability of experiencing drought, floods or temperatures above 35 °C in the critical period of maize production is greater than the respective median probability.

**Table:** 

Livelihood Capital	Livelihood Indicator	Source	Deprivation Cutoff (Based on Key Informant Interviews and Field Experience).
Natural Capital (1/5)	Agricultural Land (1/5)	[5,23,58,74,122,123]	Household is deprived if they do not have agricultural land
Physical Capital (1/5)	Area of home stead (1/15)	[5,124]	Household is deprived if the area of their home stead is below 500m
	Livestock Ownership (1/15)	[5,74]	Household is deprived if they don't own any livestock or if they share ownership of their livestock with other households or if they take care of other people's livestock for renumeration.
	Proximity to services like town, road, bank, post office, market, health centres. (1/15)	[74,122,123,125,126]	Household is deprived if it is located in a village where none of the aforementioned services are within 5 km distance.
Financial Capital (1/5)	Income (1/10)	[5,58,122,127]	Household is deprived if the household head's income falls below the minimum wage for their skill category as prescribed by the Bihar state government or household head is jobless.
	Savings (1/10)	[5,122,[126], [127], [128]]	Household is deprived if they have no savings or if their only savings is Rs. 40 at SHGs.
Human Capital (1/5)	Women's employment (1/30)	[60]	Household is deprived if no woman of working age in the household is employed or if they are seasonally employed.
	Female education (1/30)	[60,125]	Household is deprived if no female in the household of 12+ years has 6 years of schooling.
	Physical/Mental Health Condition (1/30)	[23,128]	Household is deprived if any household member is severely sick, whether physically or mentally.
	Skill Training (1/30)	[74,124]	Household is deprived if no household member has received any skill training
	Education of household head (1/30)	[58,59,123]	Household is deprived if household head does not have up to 6 years of schooling.
	Household Size (1/30)	[122,127]	If household size exceeds the average household size of the village, they are deprived.
Social Capital (1/5)	Awareness of government schemes (1/30)	[126]	Household is deprived if they are not aware of any government welfare scheme.
	Access to government schemes (1/30)	[5,60]	Household is deprived if they have not accessed any government welfare scheme.
	Caste (1/30)	[2,123,125]	Household is deprived if they belong to the scheduled caste or tribe or is muslim.
	Familial relations (1/30)	[129]	Household is deprived if they self-rated their relationship with family members as poor/average
	Neighbourhood relations (1/30)	[23]	Household is deprived if they self-rated their relationship with neighbours as poor/average
	Participation in Self Help Groups (1/30)	[125,128]	Household is deprived if no eligible woman in the household belongs to a self-help group.
